# Difference in Protein Expression Profile and Chemotherapy Drugs Response of Different Progression Stages of LNCaP Sublines and Other Human Prostate Cancer Cells

**DOI:** 10.1371/journal.pone.0082625

**Published:** 2013-12-05

**Authors:** Hui-Ping Lin, Ching-Yu Lin, Ping-Hsuan Hsiao, Horng-Dar Wang, Shih Sheng Jiang, Jong-Ming Hsu, Wai-Tim Jim, Marcelo Chen, Hsing-Jien Kung, Chih-Pin Chuu

**Affiliations:** 1 Institute of Cellular and System Medicine, National Health Research Institutes, Miaoli, County, Taiwan; 2 Translational Center for Glandular Malignancies, National Health Research Institutes, Miaoli, County, Taiwan; 3 National Institute of Cancer Research, National Health Research Institutes, Miaoli, County, Taiwan; 4 Institute of Biotechnology, National Tsing Hua University, Hsinchu City, Taiwan; 5 Department of Urology, Mackay Memorial Hospital, Taipei City, Taiwan; 6 Department of Pediatrics, Mackay Memorial Hospital, Taipei City, Taiwan; 7 Institute of Molecular and Genomic Medicine, National Health Research Institutes, Miaoli, County, Taiwan; 8 Graduate Program for Aging, China Medical University, Taichung City, Taiwan; 9 Ph.D. Program in Tissue Engineering and Regenerative Medicine, National Chung Hsing University, Taichung City, Taiwan; II Università di Napoli, Italy

## Abstract

Androgen ablation therapy is the primary treatment for metastatic prostate cancer. However, 80-90% of the patients who receive androgen ablation therapy ultimately develop recurrent tumors in 12-33 months after treatment with a median overall survival time of 1-2 years after relapse. LNCaP is a commonly used cell line established from a human lymph node metastatic lesion of prostatic adenocarcinoma. We previously established two relapsed androgen receptor (AR)-rich androgen-independent LNCaP sublines 104-R1 (androgen depleted for 12 months) and 104-R2 cells (androgen depleted for 24 months) from AR-positive androgen-dependent LNCaP 104-S cells. LNCaP 104-R1 and 104-R2 mimics the AR-positive hormone-refractory relapsed tumors in patients receiving androgen ablation therapy. Androgen treatment stimulates proliferation of 104-S cells, but causes growth inhibition and G1 cell cycle arrest in 104-R1 and 104-R2 cells. We investigated the protein expression profile difference between LNCaP 104-S vs. LNCaP 104-R1, 104-R2, PC-3, and DU-145 cells as well as examined the sensitivity of these prostate cancer cells to different chemotherapy drugs and small molecule inhibitors. Compared to 104-S cells, 104-R1 and 104-R2 cells express higher protein levels of AR, PSA, c-Myc, Skp2, BCL-2, P53, p-MDM2 S166, Rb, and p-Rb S807/811. The 104-R1 and 104-R2 cells express higher ratio of p-Akt S473/Akt, p-EGFR/EGFR, and p-Src/Src, but lower ratio of p-ERK/ERK than 104-S cells. PC-3 and DU-145 cells express higher c-Myc, Skp2, Akt, Akt1, and phospho-EGFR but less phospho-Akt and phospho-ERK. Overexpression of Skp2 increased resistance of LNCaP cells to chemotherapy drugs. Paclitaxel, androgen, and inhibitors for PI3K/Akt, EGFR, Src, or Bcl-2 seem to be potential choices for treatment of advanced prostate cancers. Our study provides rationale for targeting Akt, EGFR, Src, Bcl-2, and AR signaling as a treatment for AR-positive relapsed prostate tumors after hormone therapy.

## Introduction

According to the latest statistics in 2008 (GLOBOCAN 2008 database, version 1.2), prostate cancer is the second most frequently diagnosed cancer of men and the fifth most common cancer overall in the world. The statistics of American Cancer Society estimated that 238,590 new cases of prostate cancer will be diagnosed and approximately 29,720 people will die from prostate cancer-specific deaths in United States in 2013. Incidence of prostate cancer is increasing steadily in almost all countries [1]. Prostate cancer is diagnosed in very few people younger than 50 years. Approximately 85% of patients being diagnosed are over 65 years old [1]. Surgery is often successful for organ-confined prostate cancer. Androgen ablation therapy, proposed by Dr. Charles B. Huggins, is the primary treatment for metastatic prostate cancer. However, most prostate cancer patients receiving the androgen ablation therapy will ultimately develop recurrent, castration-resistant tumors within 1-3 years after treatment with a median overall survival time of 1-2 years after relapse [2,3]. There is no effective standard therapy for relapsed advanced prostate cancers. Chemotherapy is usually applied for treatment of metastatic hormone-refractory prostate cancer [4]. Commonly used chemotherapeutic drugs for prostate cancers include etoposide, paclitaxel, vinblastine, and mitoxantrone. Etoposide and mitoxantrone are type II topoisomerase inhibitors [4,5]. Vinblastine binds tubulin and inhibits assembly of microtubules [4]. Paclitaxel disrupts mitotic spindle assembly, chromosome segregation, and cell division. Paclitaxel also stabilizes the microtubule polymer and protects it from disassembly [4]. Chemotherapy drug treatments result in decrease of PSA, radiographic response, improvement of pain, and improvement of urinary symptoms in a sub-group of patients [4]. However, these drugs show little effect on prolonging survival [4]. Undesired side effects of these chemotherapeutic agents include toxic deaths, strokes, thrombosis, neutropenia, edema, dyspnea, malaise, and fatigue [4]. Alternative therapies are in need.

LNCaP is a commonly used cell line established from a human lymph node metastatic lesion of prostatic adenocarcinoma [6]. LNCaP cells express androgen receptor (AR) and prostate specific antigen (PSA). Previously, we cultured androgen-sensitive LNCaP 104-S cells in androgen-depleted conditions *in vitro* to mimic patients receiving androgen ablation therapy [7-9]. Most 104-S cells died after 3 months. A small population of cells named 104-R1 emerged after 10 months. These cells proliferate regularly in the absence of androgen [7-9]. Eighteen to twenty months after androgen depletion, 104-R1 cells gave rise to a faster-growing population of cells called 104-R2 cells [7-9]. During the transition of 104-S cells to 104-R1 and 104-R2 cells, the mRNA expression, protein abundance, and transcriptional activity of AR increase several folds [7-14]. Proliferation of 104-R1 and 104-R2 cells is androgen-independent but is suppressed by physiological concentrations of androgen [7-9,11-14]. Androgen treatment suppresses c-Myc and Skp2, thereby causes G1 cell cycle arrest in 104-R1 and 104-R2 cells. Our LNCaP prostate cancer progression model mimics the clinical situations in which AR-positive prostate tumors recur following androgen deprivation [13,15,16]. PC-3 and DU-145 cells belong to NCI60 and were AR-negative prostate cancer cells established from human prostatic adenocarcinoma metastatic to bone [17] and brain [18], respectively. We thus used LNCaP progression model, PC-3, and DU-145 cells in this study to characterize the difference of protein expression profile between androgen-dependent and androgen-independent prostate cancer cell. We examined the profile of 33 different proteins involved in cell cycle regulation, cell survival, Akt signaling, and epidermal growth factor receptor (EGFR) signaling in 104-S, 104-R1, 104-R2, PC-3, and DU-145 cells. We also compared the difference in sensitivity of these prostate cancer cells to treatment with several chemotherapy drugs and small molecule inhibitors to determine which drug or inhibitor is more effective to suppress the proliferation of advanced prostate cancer cells. Our observations suggested that Akt, EGFR, Src, Bcl-2, and AR signaling pathways are potential therapeutic targets for AR-positive castration-resistant prostate cancers. 

## Materials and Methods

### Cell Culture

LNCaP prostate cancer cell sublines (104-S, 104-R1, and 104-R2 cells) and PC-3 were gifts from Dr. Shutsung Liao (The University of Chicago, IL, U.S.A.). LNCaP 104-S, 104-R1, and 104-R2 cells were generated from LNCaP FGC clone (ATCC CRL-1740). These LNCaP sublines and PC-3 cells have been described in previous publications [7-12,14,19-24]. DU-145 cells were purchased from Bioresource Collection and Research Center (Hsinchu city, Taiwan). These cell lines were passaged and maintained as described previously [7-12,14,19-24]. R1881 (17β-hydroxy-17α-methylestra-4,9,11-trien-3-one) was purchased from Sigma-Aldrich (Sigma, St. Louis, MO, U.S.A.).

### Chemicals

EGFR inhibitor Gefitinib (Iressa, ZD-1839) and Src inhibitor Saracatinib (AZD0530) were purchased from Selleckchem (Houston, TX, U.S.A.). BCl-2 inhibitor ABT 737 (CAS 852808-04-9) was purchased from Santa Cruz (Santa Cruz, CA, U.S.A.). Other compounds were purchased from Sigma.

### Cell Proliferation Assay

Relative cell number was analyzed by measuring DNA content of cell lysates with the fluorescent dye Hoechst 33258 (Sigma) as described previously [10,14,19,20,22-25]. The mean and standard deviation represented the average of octuplicate (8 wells) of a representative experiment. 

### Flow Cytometric Analysis

Cells were seeded at a density of 5 x 10^5^ cells in 10-cm dishes in 10 mL media for 24 hr. After 96 hr of culture in the presence of various concentrations of androgen, cells were removed with trypsin and fixed in 70% ethanol in PBS overnight at -20°C. Fixed cells were washed with PBS, treated with 0.1 mg/mL RNase A in PBS for 30 min, and then suspended in 50 µg/mL propidium iodide in PBS. Cell cycle profiles and distributions were determined by flow cytometric analysis of cells using a BD Facscan flow cytometer (BD Biosciences, San Jose, CA, U.S.A.). Cell cycle distribution was analyzed using ModFit LT software (Verity Software House, Topsham, ME, U.S.A.) as described [8,9,14,20,22,23,25].

### Real-Time Quantitative Polymerase Chain Reaction

RNA was isolated and specific mRNAs were quantified as described [10-12,19,21,26]. The sequences of primers and probes for androgen receptor (AR) and glyceraldehyde-3-phosphate dehydrogenase (GAPDH) were described previously [9,12,26]. Primer and probe sequences used for PSA quantification were described by Gelmini et al [27]. All transcript levels were normalized to GAPDH levels in each sample.

### Western Blotting Analysis

Cells samples were lysed in SDS lysis buffer (240 mM Tris-acetate, 1% SDS, 1% glycerol, 5mM EDTA pH 8.0, 0.1mM dithiothreitol) containing the protease inhibitors 1 mM 4-(2-aminoethyl)benzenesulfonyl fluoride (AEBSF), 0.8 μM aprotinin, 40 μM bestatin, 14 μM E-64, 20μM leupeptin, 15μM Pepstatin A (Sigma-Aldrich, P8340) and the phosphatase inhibitors cantharidin, bromotetramisole and microcystin LR (Sigma-Aldrich, P2850). Proteins were separated on 8-12% SDS-PAGE gels and expression levels of proteins were determined by Western blotting using the following antibodies: Akt2, β-actin and GAPDH were from Novus (Littleton, CO, U.S.A.). Total Akt, phospho-Akt Ser473, phospho-Akt Thr308, phodpho-p42/44 MAPK Thr202/Tyr204, EGFR, p-MDM2, p53, Src, phospho-Src Tyr527, Rb, and p-Rb Ser807/811 were from Cell Signaling (Danvers, MA, U.S.A.). Fatty acid synthase (FAS), AR and c-Myc antibodies were purchased from Epitomics (Burlingame, CA, U.S.A.). Skp2, p21, and p27 antibodies were purchased from Santa Cruz (Santa Cruz, CA, U.S.A.). PSA was from DAKO (Elostrup, Denmark). Bcl-2 was from BD (BD Biosciences, San Jose, CA, U.S.A.). Bad, MDM2, Akt1, p42/44 MAPK, phospho-EGFR Tyr1173, phospho-EGFR Tyr1148, phospho-EGFR Tyr1086, phospho-EGFR Tyr1069, phospho-EGFR Tyr1045, and phospho-EGFR Tyr 845 were from Millipore (Billerica, MA, U.S.A.). α-tubulin was from Sigma. β-actin, α-tubulin, and GAPDH were used as loading control. Horseradish peroxidase-conjugated anti-rabbit and anti-mouse IgG secondary antibodies were from Santa Cruz. The signal of horseradish peroxidase labeled secondary antibodies was detected by enhanced chemoluminescence reaction (ECL Western Blotting detection kit) (PerkinElmer, Waltham, MA, U.S.A.). GAPDH, α-tubulin, and β-actin were used as loading controls. Intensity of bands for different proteins was quantified with EPSON stylus TX130 using UN-SCAN-IT gel 6.1 software.

### Sequencing of AR ligand binding domain in LNCaP sublines

Total RNA was isolated with RNeasy Mini Kit (Qiagen, Venlo, Hilden, Germany). cDNA was synthesized from total RNA using RevertAid H Minus First Strand cDNA Synthesis Kit (Fermentas, Waltham, Massachusetts, U.S.A.). The coding sequence for ligand-binding domain of AR was amplified with KOD-plus kit (TOYOBO, Osaka, Japan), using the primer pair 5’-ctgaaactacaggaggaagg-3’(forward) and 5’-tgcagaggagtagtgcagag-3’(reverse). Amplification was performed using a touch-down thermal protocol: 8 cycles of 94°C for 15 s, 55 to 47°C for 30 s (decreased the annealing temperature 1°C per cycle), 68°C for 1 min, followed by 35 cycles of 94°C for 15s, 47°C for 30s, 68°C for 1 min, and a final extension step at 68°C for 3min. PCR products were gel-purified (FAVORGEN, Ping-Tung, Taiwan) and direct sequencing on a DNA Sequencer. The sequencing data was deposited to GenBank (submission ID: 1664456; BankIt1664456 Seq1 KF720403; BankIt1664456 Seq2 KF720404; BankIt1664456 Seq3 KF720405).

### Data Analysis

Data are presented as the mean +/– SD of at least three experiments or are representative of experiments repeated at least three times. Student’s t test (two-tailed, unpaired) was used to evaluate the statistical significance of results from proliferation assay experiments. An Excel add-in program ED50V10 was used for calculating half maximal inhibitory concentration (IC_50_).

## Results

### Androgenic response of LNCaP sublines

**Figure 1:**
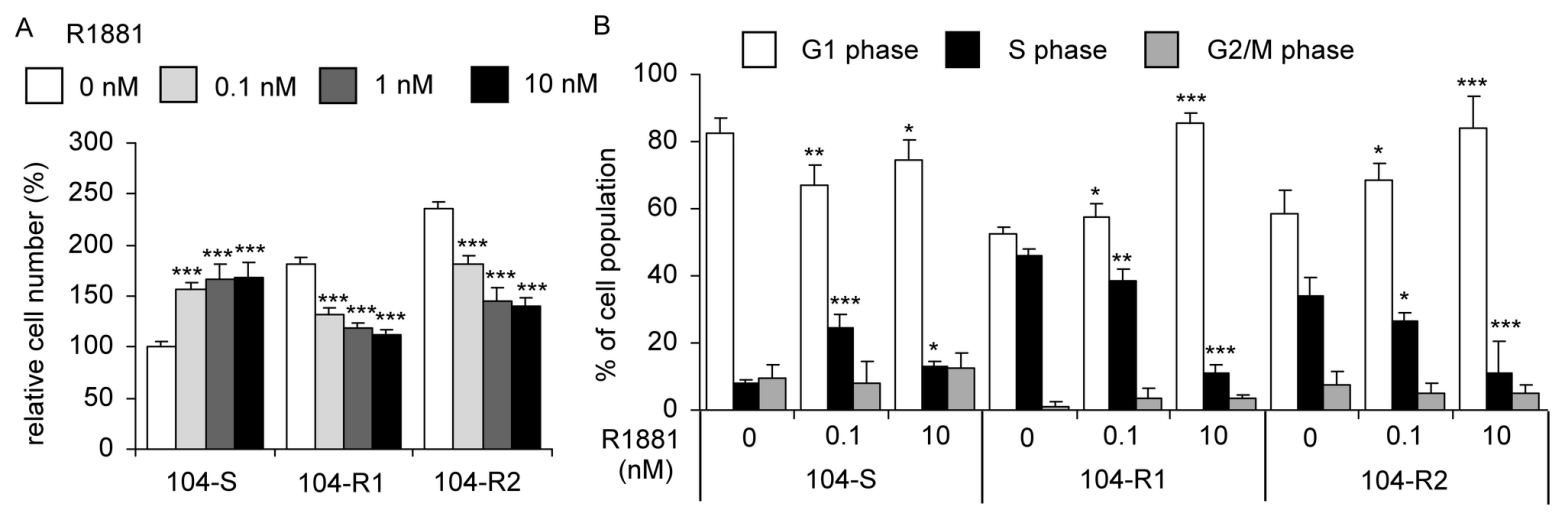
Androgen effect on proliferation and cell cycle of LNCaP sublines. Proliferation of LNCaP 104-S, 104-R1, and 104-R2 cells growing in 10% CS-FBS DMEM plus 0, 0.1, 1, or 10 nM synthetics androgen R1881 for 96 hours was determined by 96-well proliferation measuring total DNA content per well using Hoechst 33258 fluorescence. Relative cell numbers were normalized to that of 104-S cells without R1881 treatment. Triple asterisks (***) represents cell number is statistically significantly different (P<0.001) compared to the same cell type without R1881 treatment. Columns represent mean for 24 replicates; bars represent standard deviation. (B) Cell cycle distribution of LNCaP 104-S, 104-R1, and 104-R2 cells treated with 0, 0.1, and 10 nM R1881 for 96 hours was determined by flow cytometry. Asterisk (*), double asterisks (**), and triple asterisks (***) indicate statistically significant difference of *P* < 0.05, *P* < 0.01, and *P* < 0.001, respectively, as compared to the same cell type without R1881 treatment.

The main difference between LNCaP 104-R1 or 104-R2 cells with their parental 104-S cells is their response to androgen treatment. Treating LNCaP 104-S cells with physiological concentration of androgen (0.1, 1, 10 nM R1881) [28] stimulated cellular proliferation. However, androgen treatment suppressed proliferation of 104-R1 and 104-R2 cells (Figure 1A). In the absence of androgen, the growth rate of 104-R1 and 104-R2 cells was 1.8-2.3 folds higher than that of 104-S cells (Figure 1A). Androgen treatment (0.1, 10 nM R1881) increased the cell population in S phase and decreased the cell population in G1 phase in 104-S cells. However, androgen decreased cell population in S phase and caused G1 cell cycle arrest in both 104-R1 and 104-R2 cells (Figure 1B). The IC_50_ of R1881 calculated to suppress 104-R1 and 104-R2 cells was 14.9 and 13.2 nM, respectively. PC-3 and DU-145 cells do not express AR and do not respond to androgen treatment, we thus did not include PC-3 and DU-145 cells in these experiments.

### Expression of AR and PSA mRNA in LNCaP sublines

**Figure 2:**
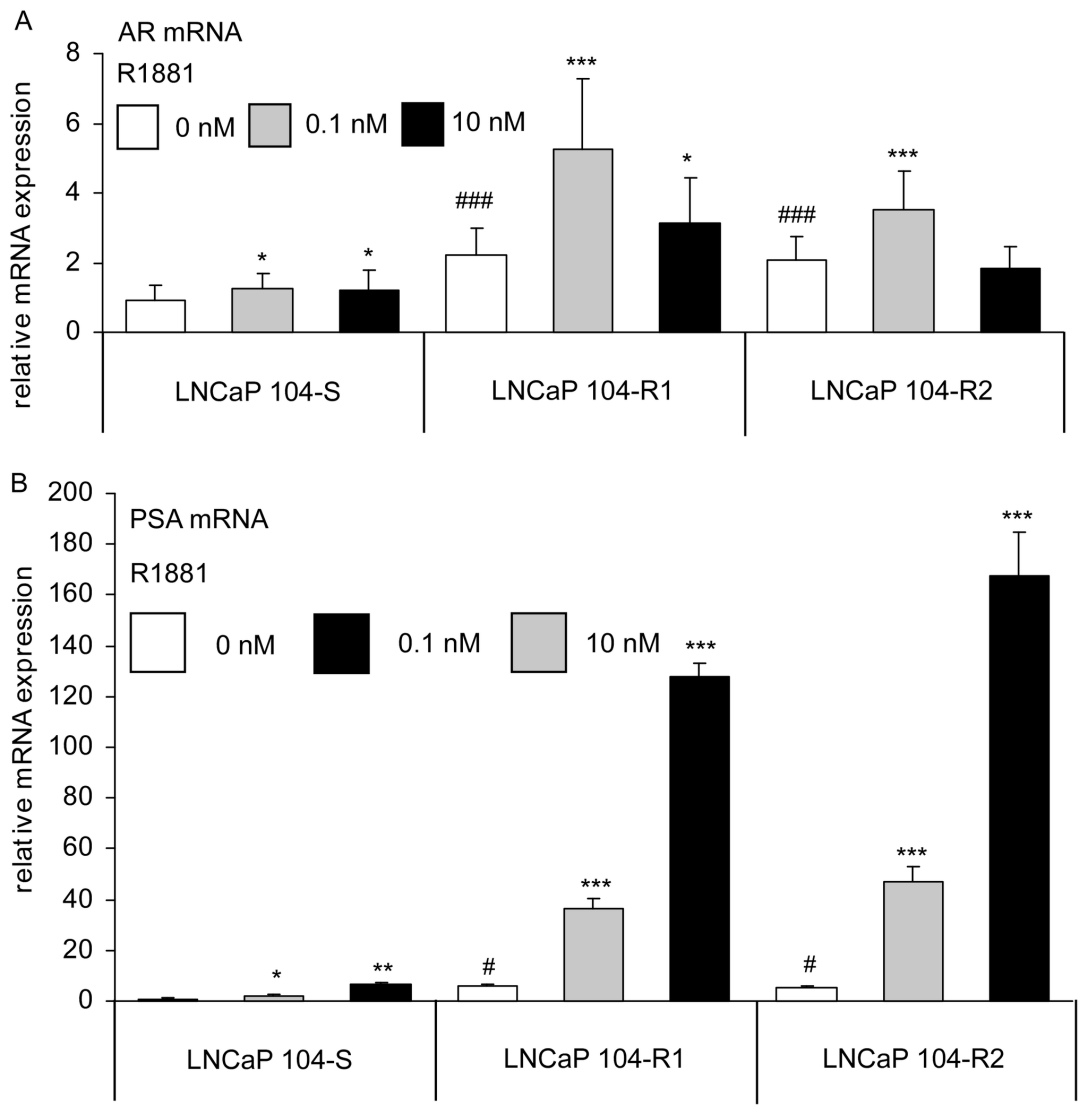
The mRNA expression of AR and PSA in LNCaP sublines. Expression of AR (A) and PSA (B) mRNA was detected with quantitative real-time PCR in 104-S, 104-R1, and 104-R2 cells treated with 0, 0.1, and 10 nM of R1881 for 96 hours. Asterisk (*), double asterisks (**), and triple asterisks (***) indicate statistically significant difference of *P* < 0.05, *P* < 0.01, and *P* < 0.001, respectively, as compared to the same cell type without R1881 treatment. Number sign (#) and (###) indicate statistically significant difference of *P* < 0.05 and *P* < 0.001, respectively, for 104-R1 or 104-R2 cells as compared to 104-S cells in the absence of R1881.

Similar to our previous observations [7-9,14], 104-R1 and 104-R2 cells express higher AR mRNA than 104-S cells when androgen is absent (Figure 2A). In addition, we observed that 0.1 nM R1881 increased expression of AR mRNA in all 104-S, 104-R1, and 104-R2 cells, while 10 nM R1881 only increased AR mRNA in 104-S and 104-R1 cells (Figure 2A). Transcriptional activity of AR in 104-S, 104-R1, and 104-R2 cells was compared by examining the androgenic induction of mRNA expression of the AR target gene prostate specific antigen (PSA). PSA mRNA levels increased with R1881 treatment dose-dependently but were much higher in 104-R1 and 104-R2 cells than those in 104-S cells (Figure 2B). Treating 104-S cells with 0.1 nM R1881 caused a 2.3 fold increase of PSA mRNA. However, R1881 at 0.1 nM caused an approximately 6.3 and 9.0 fold increase of PSA mRNA in 104-R1 and 104-R2 cells, respectively. Treatment with 10 nM R1881 caused a 6.8 fold increase of PSA mRNA in 104-S cells, but caused an approximately 22.1 and 32.0 fold increase of PSA mRNA in 104-R1 and 104-R2 cells, respectively (Figure 2B). When R1881 was absent, the PSA mRNA level was higher in 104-R1 and 104-R2 cells than that in 104-S cells. PC-3 and DU-145 cells do not express AR and do not respond to androgen treatment, we thus did not include PC-3 and DU-145 cells in these experiments. 

### AR ligand binding domain in LNCaP sublines

**Figure 3:**
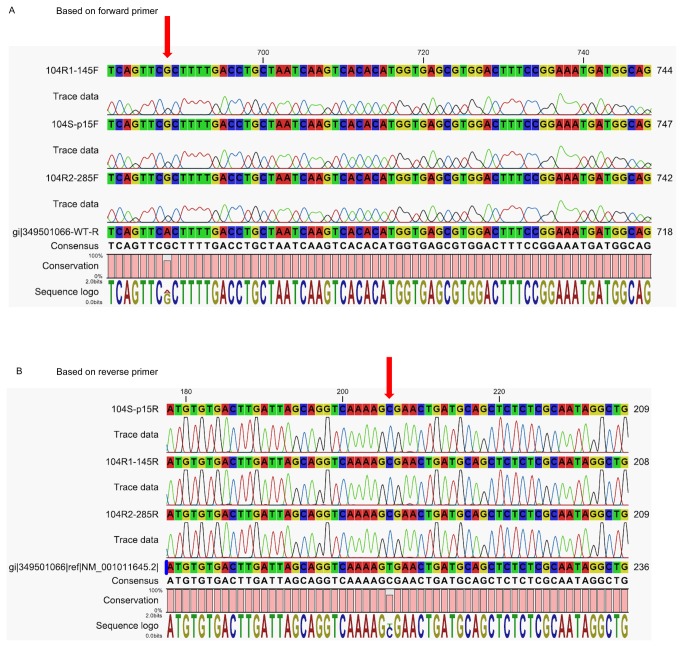
Sequence of AR ligand binding domain (LBD) in LNCaP sublines. Sequence of LBD of AR in 104-S, 104-R1, and 104-R2 cells based on forward primer (A) and reverse primer (B) was shown. Red arrow indicated the point mutation T877A on LBD of AR in LNCaP cells.

LNCaP cells express a mutant AR (T877A) that displays relaxed ligand binding specificity [29,30]. As 104-R1 and 104-R2 cells showed greater androgenic response (Figure 2B), we determined if there are additional mutations in 104-R1 and 104-R2 cells other than T877A. Sequencing of cDNA from LNCaP 104-S, 104-R1, and 104-R2 cells (Figure 3A, 3B) suggested that all three LNCaP sublines contain the mutation T877A on AR. There was no additional mutation in 104-R1 and 104-R2 cells as compared to their parental 104-S cells. The greater androgenic response may be due to higher AR expression level in 104-R1 and 104-R2 cells (Figure 2B).

### Profiling of cell cycle regulation proteins in LNCaP sublines, PC-3, and DU-145 cells

**Figure 4:**
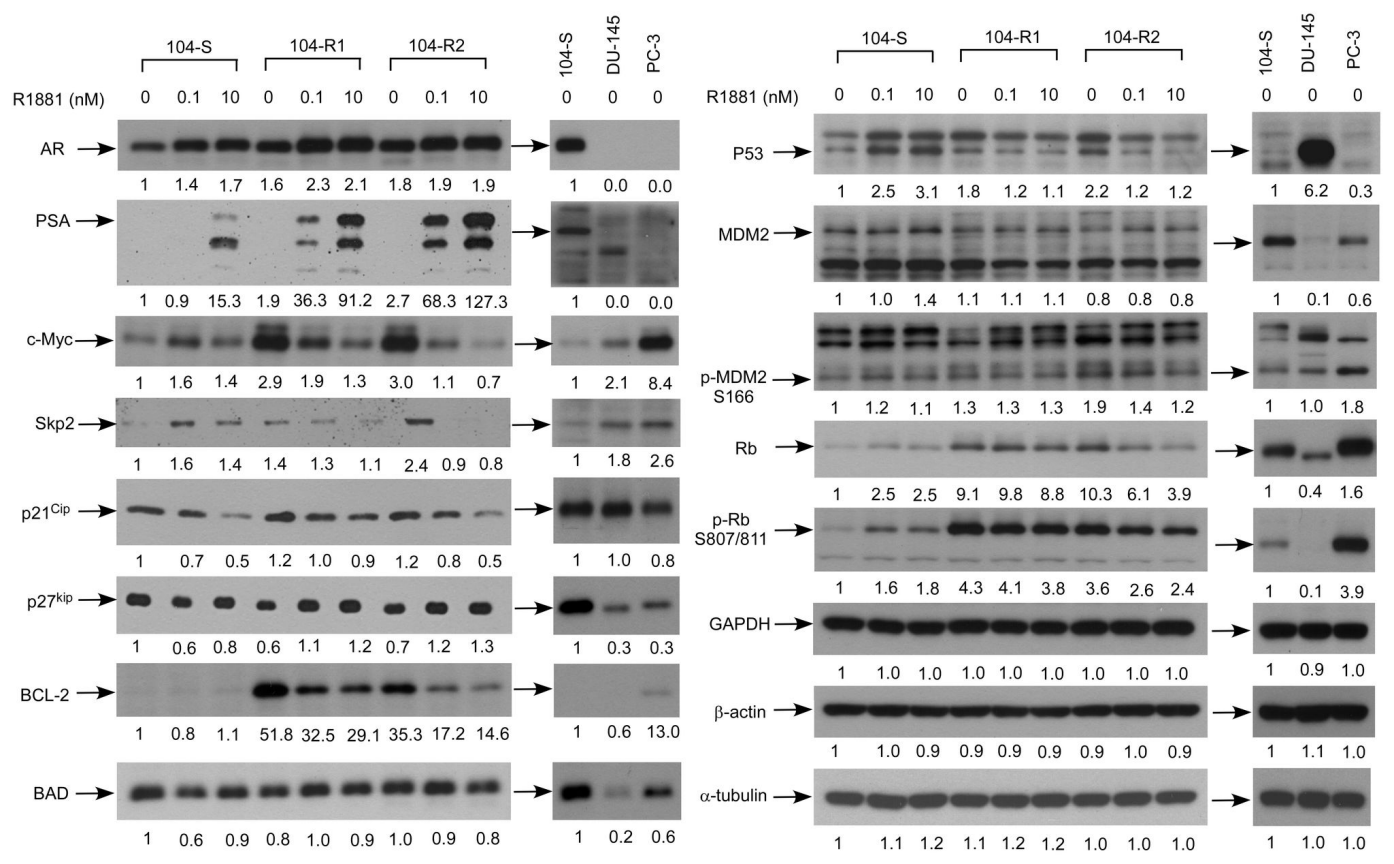
Effect of androgen treatment on protein abundance of AR, PSA, and cell cycle regulators in LNCaP sublines, DU-145, and PC-3 cells. Protein expression of AR, PSA, c-Myc, Skp2, p21^Cip1^, p27^Kip1^, Bcl-2, Bad, p53, MDM2, phospho-MDM2 Ser166, Rb, phospho-Rb Ser807/811, GAPDH, α-tubulin, and β-actin in LNCaP 104-S, 104-R1, and 104-R2 cells treated with 0, 0.1, or 10 nM R1881 for 96 hours were assayed by Western blotting. GAPDH, α-tubulin, and β-actin were used as loading control. Experiments were repeated three times. These proteins were also examined in PC-3 and DU-145 cells in the absence of androgen. Numbers represent quantification of bands of individual protein quantified by ImageJ and UN-SCAN-IT gel 6.1 software.

Protein expression level of AR is higher in LNCaP 104-R1 and 104-R2 as compared to LNCaP 104-S cells in the absence of androgen. Treatment with both 0.1 and 10 nM synthetic androgen R1881 slightly increased AR proteins in all LNCaP sublines (Figure 4). DU-145 and PC-3 cells do not express AR (Figure 4). All three LNCaP sublines but not DU-145 and PC-3 cells express PSA proteins. Androgen treatment induced production of PSA protein. PSA protein level was much higher in 104-R1 and 104-R2 cells as compared to 104-S cells when being treated with 10 nM R1881. Treatment with 0.1 nM R1881 only induced PSA expression in 104-R1 and 104-R2 cells but not 104-S cells. The induction fold of PSA protein by androgen treatment was similar to the induction fold of PSA mRNA (Figure 2B, Figure 4). 

When androgen is absent, 104-R1 and 104-R2 cells expressed higher levels of c-Myc, Skp2, BCL-2, P53, phospho-MDM2 Ser166, Rb, and phospho-Rb Ser807/811 but less p27 ^Kip^ proteins as compared to 104-S cells. Androgen treatment increased protein expression of Skp2, c-Myc, Rb, and phospho-Rb Ser 807/811, and P53 in 104-S cells but decreased the expression of these proteins in 104-R1 and 104-R2 cells. Expression of P27^Kip^ was reduced by androgen in 104-S cells but was induced in 104-R1 and 104-R2 cells. Androgen treatment decreased P21^Cip^ abundance in all LNCaP sublines. BCL-2 protein expression in 104-R1 and 104-R2 cells was high but BCL-2 protein was barely detectable in 104-S cells. Androgen treatment decreased BCL-2 in 104-R1 and 104-R2 cells. MDM2 in 104-S cells was slightly increased by androgen but was not affected by androgen in 104-R1 and 104-R2 cells. BAD protein expression was not affected by androgen treatment in 104-R1 and 104-R2 LNCaP sublines (Figure 4). 0.1 nM R1881 slightly decreased BAD protein in 104-S cells. DU-145 and PC-3 cells expressed higher c-Myc and Skp2 but lower p27^Kip^ as compared to 104-S cells when androgen was absent. When compared to 104-S cells, DU-145 cells expressed higher p53 and lower BAD protein. PC-3 cells expressed higher BCl-2, phospho-MDM2 S166, Rb, and phospho-Rb S807/811 but lower BAD, P53, and MDM2 as compared to 104-S cells.

### Profiling of Akt and EGFR signaling proteins in LNCaP sublines, PC-3, and DU-145 cells

**Figure 5:**
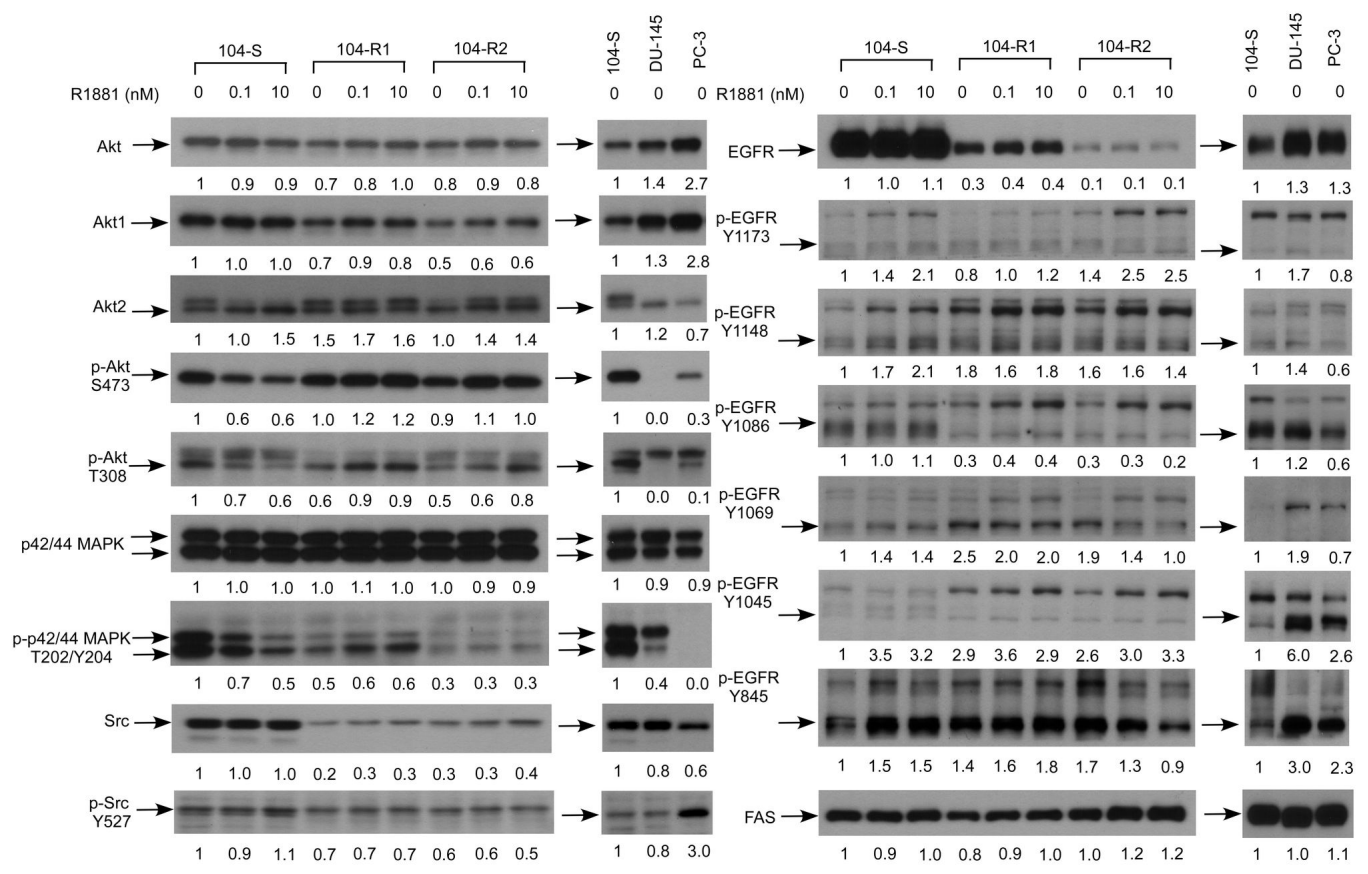
Effect of androgen treatment on protein abundance of AR, PSA, and cell cycle regulators in LNCaP sublines, DU-145, and PC-3 cells. Protein expression of total Akt, Akt1, Akt2, phospho-Akt Ser473, phospho-Akt Thr308, p42/44 MAPK, phospho-p42/44 MAPK Thr202/Tyr204, EGFR, and different phosphorylation site of tyrosine (Tyr1173, Tyr1148, Tyr1086, Tyr1069, Tyr1045, and Tyr845) in LNCaP 104-S, 104-R1, and 104-R2 cells treated with 0, 0.1, or 10 nM R1881 for 96 hours were assayed by Western blotting. Same GAPDH, α-tubulin, and β-actin as shown in Figure 3 were used as loading control. These proteins were also examined in PC-3 and DU-145 cells in the absence of androgen. Experiments were repeated three times. Numbers represent quantification of bands of individual protein quantified by ImageJ.

Akt and epidermal growth factor receptor (EGFR) signaling plays important roles regulating the proliferation and progression of prostate cancer. Compared to 104-S cells, 104-R1 and 104-R2 cells expressed higher protein levels of Akt2 and phosphorylation of tyrosines on EGFR (Tyr 1173, Tyr 1148, Tyr 1069, Tyr 1045, and Tyr 845) but expressed lower protein levels of Akt, Akt1, phospho-EGFR Tyr 1086, phospho-Akt Thr308, phospho-p42/44 MAPK Thr202/Tyr 204, Src, phospho-Src Tyr527, and EGFR (Figure 5). Androgen treatment increased protein expression of Akt2 in all three sublines as well as phospho-EGFR Tyr 1148, Tyr 1069, Tyr 1045, and Tyr 845 in 104-S cells. Androgen decreased phospho-Akt Ser473, phospho-Akt Thr308, and phospho-p42/44 MAPK Thr202/Tyr 204 in 104-S cells but not in 104-R1 and 104-R2 cells. Androgen slightly increased phosphorylation of Akt (Thr308 and Ser473) in 104-R1 and 104-R2 cells. Protein expression of total Akt, Akt1, p42/44 MAPK, and EGFR was not affected by androgen treatment. Compared to 104-S cells, DU-145 and PC-3 cells expressed higher Akt and Akt1 and similar level of p42/44 MAPK. However, the protein level of phospho-Akt T308 and S473 as well as phospho-p42/44 MAPK was very low in DU-145 and PC-3 cells. Although PC-3 cells expressed less Src protein, phospho-Src Tyr527 was relatively high in PC-3 cells as compared to 104-S cells. Protein level of phospho-EGFR Tyr1045 and Tyr845 was higher in DU-145 and PC-3 as compared to 104-S cells.

We noticed an interesting finding when we normalized the phospho-protein of Akt, EGFR, and Src by their total protein abundance. 104-R1 and 104-R2 cells express relatively high ratio of phospho-Akt/Akt, phospho-Src/Src, phospho-EGFR/EGFR, but lower ratio of phospho-ERK/ERK (Table 1). Under growing condition (0.1 nM R1881 for 104-S; 0 nM R1881 for 104-R1 and 104-R2 cells), ratio of phospho-Akt/Akt, phospho-Src/Src, and phospho-EGFR/EGFR in 104-R1 and 104-R2 cells was 1.6-2 fold, 2.2-3.9, and 1.7-10 fold higher. The ratio of p-Akt/Akt was not affected very much by androgen in all three LNCaP sublines. Androgen decreased ratio of p-Src/Src in 104-R1 and 104-R2 cells but not 104-S cells. Androgen slightly increased ratio of p-EGFR/EGFR in 104-S cells. Androgen decreased ratio of p-EGFR/EGFR on Tyr1148, Tyr1069, and Tyr 1045 in 104-R1 and Tyr1069 and Tyr845 in 104-R2 cells. Androgen increased the ratio of phospho-EGFR Tyr1173/EGFR and phospho-EGFR Tyr1045/EGFR in 104-R2 cells. Compared to 104-S, DU-145 and PC-3 cells expressed very low phospho-Akt/Akt and phospho-ERK/ERK ratio but higher phospho-EGFR Tyr1045/EGFR and phospho-EGFR Tyr845/EGFR ratio. PC-3 cells also expressed relatively low ratio of phospho-EGFR/EGFR of Tyr1173, Tyr1148, Tyr1086, Tyr 1069 but very high ratio of phospho-Src/Src as compared to 104-S cells. 

**Table 1:** Ratio of phospho-Akt/Akt, phospho-Src/Src, phospho-EGFR/EGFR, and phospho-ERK/ERK for LNCaP 104-S, 104-R1, 104-R2, PC-3, and DU-145 cells.

		**LNCaP 104-S**			**LNCaP 104-R1**			**LNCaP104-R2**			**DU-145**	**PC-3**
	**R1881 (nM)**	**0**	**0.1**	**10**	**0**	**0.1**	**10**	**0**	**0.1**	**10**	**0**	**0**
**Akt**	**S473**	**1**	**0.7**	**0.7**	**1.4**	**1.5**	**1.2**	**1.1**	**1.2**	**1.3**	**0.0**	**0.1**
	**T308**	**1**	**0.8**	**0.7**	**0.9**	**1.1**	**0.9**	**0.6**	**0.7**	**1.0**	**0.0**	**0.0**
**Src**	**Y527**	**1**	**0.9**	**1.1**	**3.5**	**2.3**	**2.3**	**2.0**	**2.0**	**1.3**	**1.0**	**5.0**
**EGFR**	**Y1173**	**1**	**1.4**	**1.9**	**2.7**	**2.5**	**3.0**	**14.0**	**25.0**	**25.0**	**1.3**	**0.6**
	**Y1148**	**1**	**1.7**	**1.9**	**6.0**	**4.0**	**4.5**	**16.0**	**16.0**	**14.0**	**1.1**	**0.5**
	**Y1086**	**1**	**1.0**	**1.0**	**1.0**	**1.0**	**1.0**	**3.0**	**3.0**	**2.0**	**0.9**	**0.5**
	**Y1069**	**1**	**1.4**	**1.3**	**8.3**	**5.0**	**5.0**	**19.0**	**14.0**	**10.0**	**0.7**	**0.5**
	**Y1045**	**1**	**3.5**	**2.9**	**9.7**	**9.0**	**7.3**	**26.0**	**30.0**	**33.0**	**4.6**	**2.0**
	**Y845**	**1**	**1.5**	**1.4**	**4.7**	**4.0**	**4.5**	**17.0**	**13.0**	**9.0**	**2.3**	**1.8**
**ERK1/2**	**T202/Y204**	**1**	**0.7**	**0.5**	**0.5**	**0.5**	**0.6**	**0.3**	**0.1**	**0.1**	**0.4**	**0.0**

Quantification of protein from Figure 4 and Figure 5 was used for calculation of ratio. Values in Table 2 indicate the ratio of phospho-protein over total protein abundance.

### Pattern of protein expression profile in three LNCaP sublines, DU-145, and PC-3

**Figure 6:**
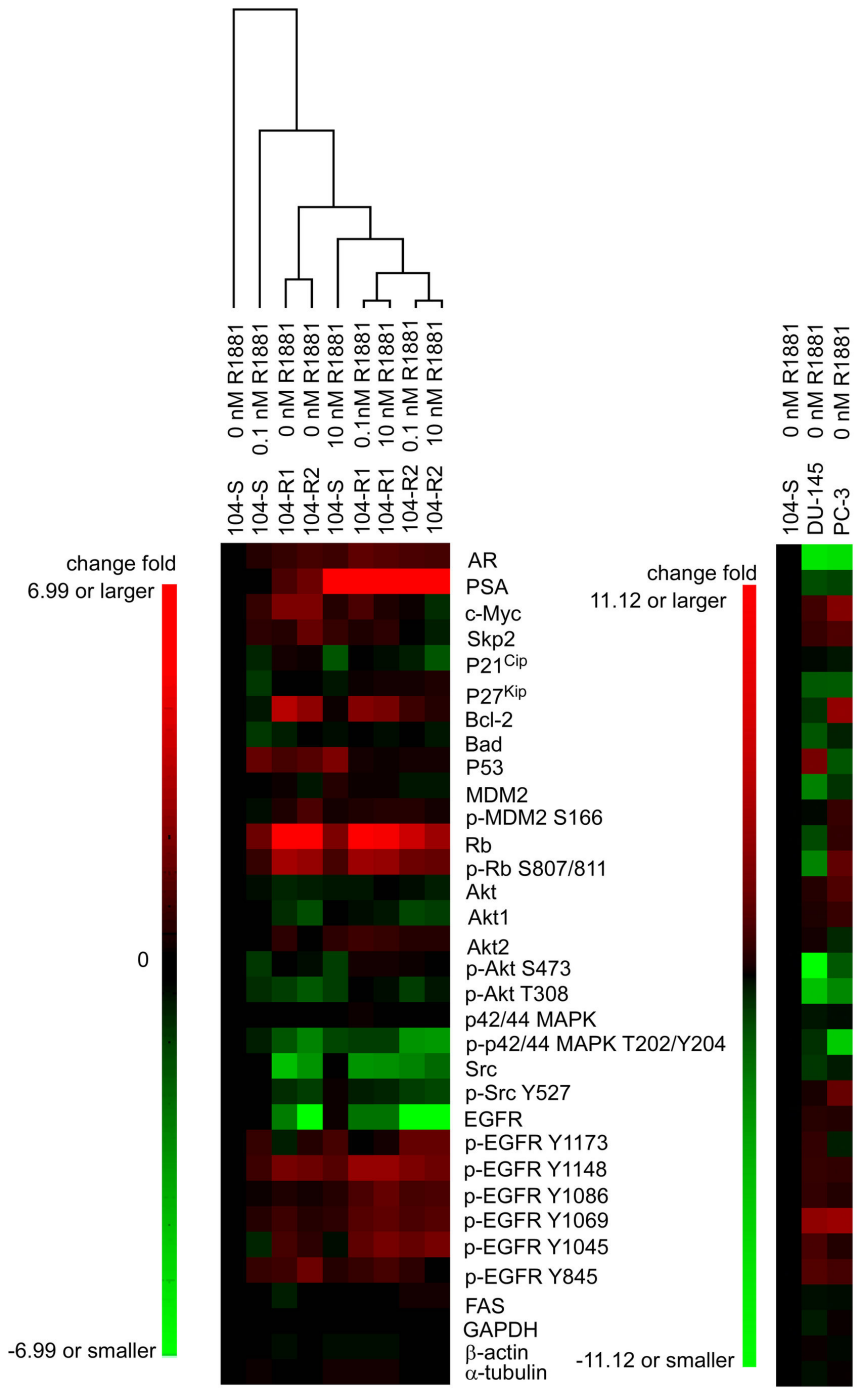
Heat map of signaling proteins abundance in LNCaP cells in the presence or absence of androgen as well as in PC-3 and DU-145 cells. Protein expression profile of LNCaP 104-S, 104-R1, and 104-R2 cells treated with 0, 0.1, or 10 nM R1881 for 96 hours as well as in DU-145 and PC-3 cells in the absence of androgen assayed in Figure 3 and Figure 4 was combined and generated into two heat map diagrams using Cluster 3.0 TreeView (http://genome-www.standford.edu). Red and green color represents the increase and decrease of protein abundance, respectively. Brighter color indicates larger change of protein abundance.

Profile of protein changes under treatment of 0, 0.1, and 10 nM R1881 in 104-S, 104-R1, and 104-R2 cells was summarized in heat map in Figure 6. It is interesting to notice that the pattern of protein expression of 104-S cells treated with 10 nM R1881 was more similar to 104-R1 and 104-R2 cells treated with androgen (0.1 and 10 nM R1881). The pattern of protein expression of 104-S cells without androgen treatment was very different from all other samples being tested (Figure 6).

### Sensitivity of LNCaP sublines, DU-145, and PC-3 cells to chemotherapy drugs

**Figure 7:**
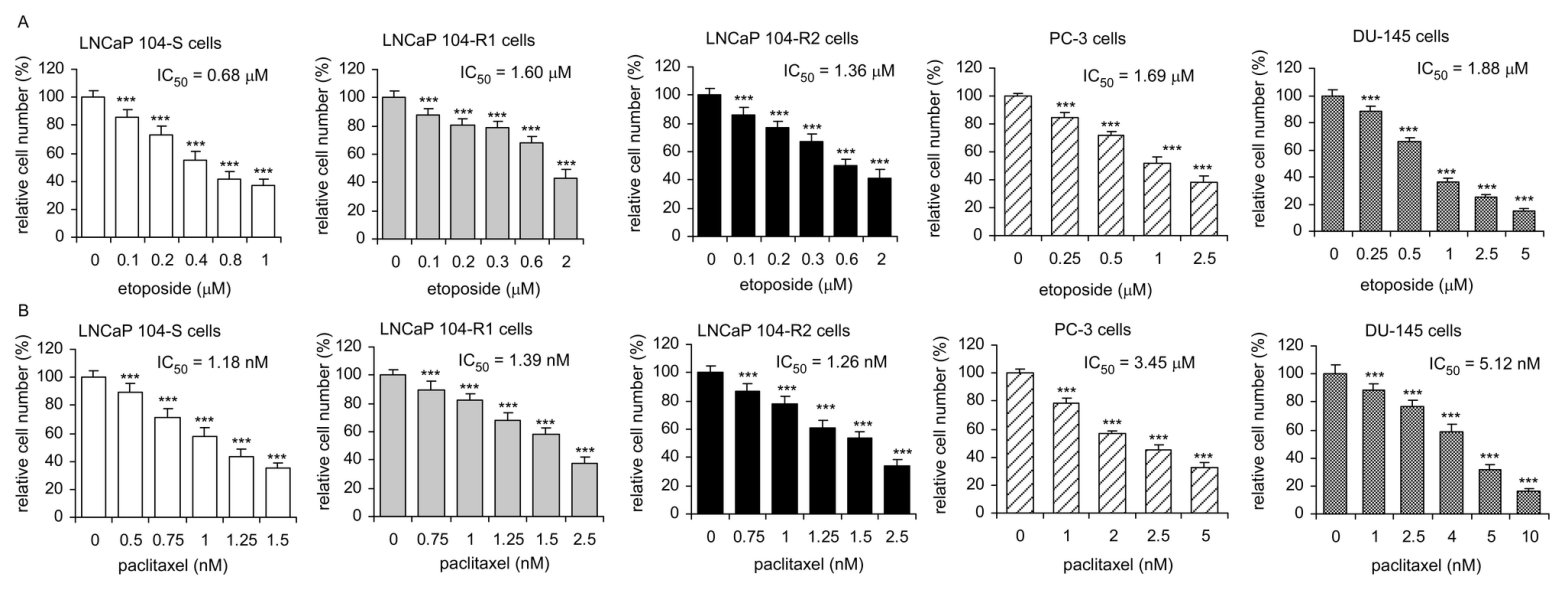
Effect of etoposide and paclitaxel on proliferation of LNCaP, PC-3, and DU-145 cell lines. LNCaP 104-S, 104-R1, 104-R2, PC-3, and DU-145 cells were treated with increasing concentrations of etoposide (A) or paclitaxel (B) for 72 hrs. Relative cell number of LNCaP cells was determined by Hoechst dye 33258-based 96-well proliferation assay as described in Materials and Methods. Cell numbers were normalized to control (dimethylsulfoxide) of each cell line. Triple asterisks (***) represent statistically significant difference *p* <0.001 between the treated group and the control (no treatment) group.

**Figure 8:**
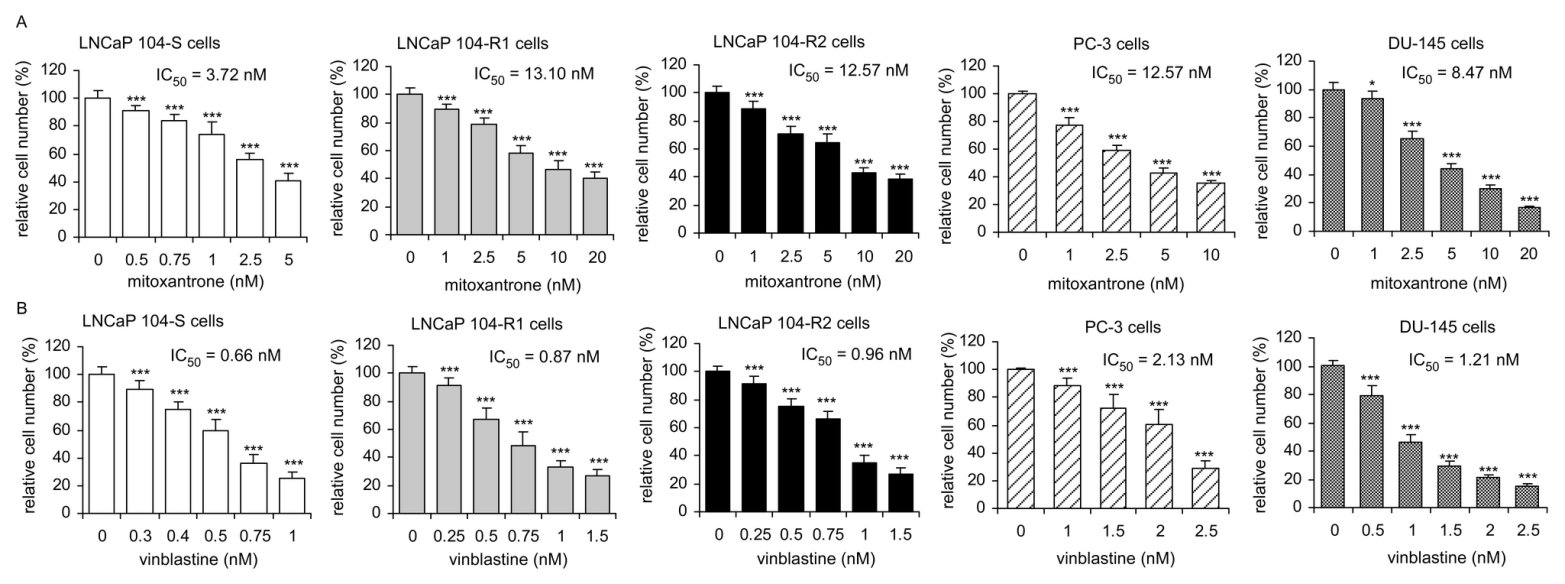
Effect of mitoxantrone and vinblastine on proliferation of LNCaP, PC-3, and DU-145 cell lines. LNCaP 104-S, 104-R1, 104-R2, PC-3, and DU-145 cells were treated with increasing concentrations of mitoxantrone (A) or vinblastine (B) for 72 hrs. Relative cell number of LNCaP cells was determined by Hoechst dye 33258-based 96-well proliferation assay as described in Materials and Methods. Cell numbers were normalized to control (dimethylsulfoxide) of each cell line. Triple asterisks (***) represent statistically significant difference *p* <0.001 between the treated group and the control group.

We next determined the growth response of LNCaP 104-S, 104-R1, 104-R2, DU-145, and PC-3 cells to four commonly used chemotherapy drugs of prostate cancers. The IC_50_ of paclitaxel for all three LNCaP sublines was similar (Figure 7 and Figure 8). The sensitivity of 104-R1 and 104-R2 cells to etoposide, mitoxantrone, and vinblastine, was similar. 104-R1 and 104-R2 cells were more resistant to treatment of etoposide, mitoxantrone, and vinblastine compared to 104-S cells (Table 2). DU-145 and PC-3 cells were more resistant to all four chemotherapy drugs as compared to 104-S cells. 

**Table 2:** Ratio of IC_50_ for LNCaP 104-R1, 104-R2, PC-3, and DU-145 cells compared to 104-S cells.

	**LNCaP 104-S**	**LNCaP 104-R1**	**LNCaP 104-R2**	**PC-3**	**DU-145**
**etoposide**	**1**	**2.35**	**2.00**	**2.49**	**2.76**
**paclitaxel**	**1**	**1.18**	**1.07**	**2.92**	**3.43**
**mitoxantrone**	**1**	**3.52**	**3.38**	**3.38**	**2.28**
**vinblastine**	**1**	**1.32**	**1.45**	**3.23**	**1.83**
**AG1478**	**1**	**1.84**	**1.41**	**0.50**	**1.69**
**Gefitinib**	**1**	**1.09**	**0.99**	**1.79**	**1.32**
**LY294002**	**1**	**1.49**	**1.33**	**2.95**	**3.73**
**Saracatinib**	**1**	**1.11**	**0.90**	**1.87**	**0.77**
**BCl-2 inhibitor**	**1**	**1.15**	**1.03**	**0.57**	**1.12**

**Figure 9:**
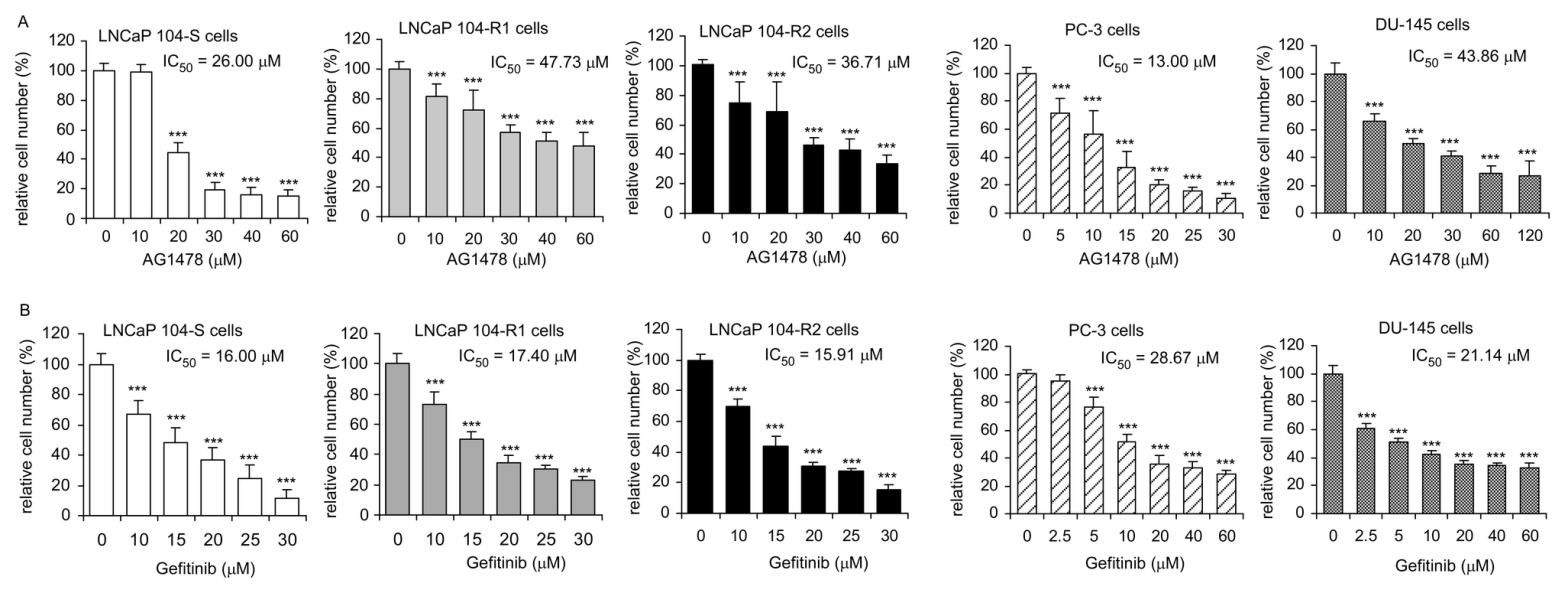
Effect of AG1478 and Gefitinib on proliferation of LNCaP, PC-3, and DU-145 cell lines. LNCaP 104-S, 104-R1, 104-R2, PC-3, and DU-145 cells were treated with increasing concentrations of AG1478 (A) or Gefitinib (B) for 72 hrs. Relative cell number of LNCaP cells was determined by Hoechst dye 33258-based 96-well proliferation assay as described in Materials and Methods. Cell numbers were normalized to control (dimethylsulfoxide) of each cell line. Triple asterisks (***) represent statistically significant difference *p* <0.001 between the treated group and the control group.

**Figure 10:**
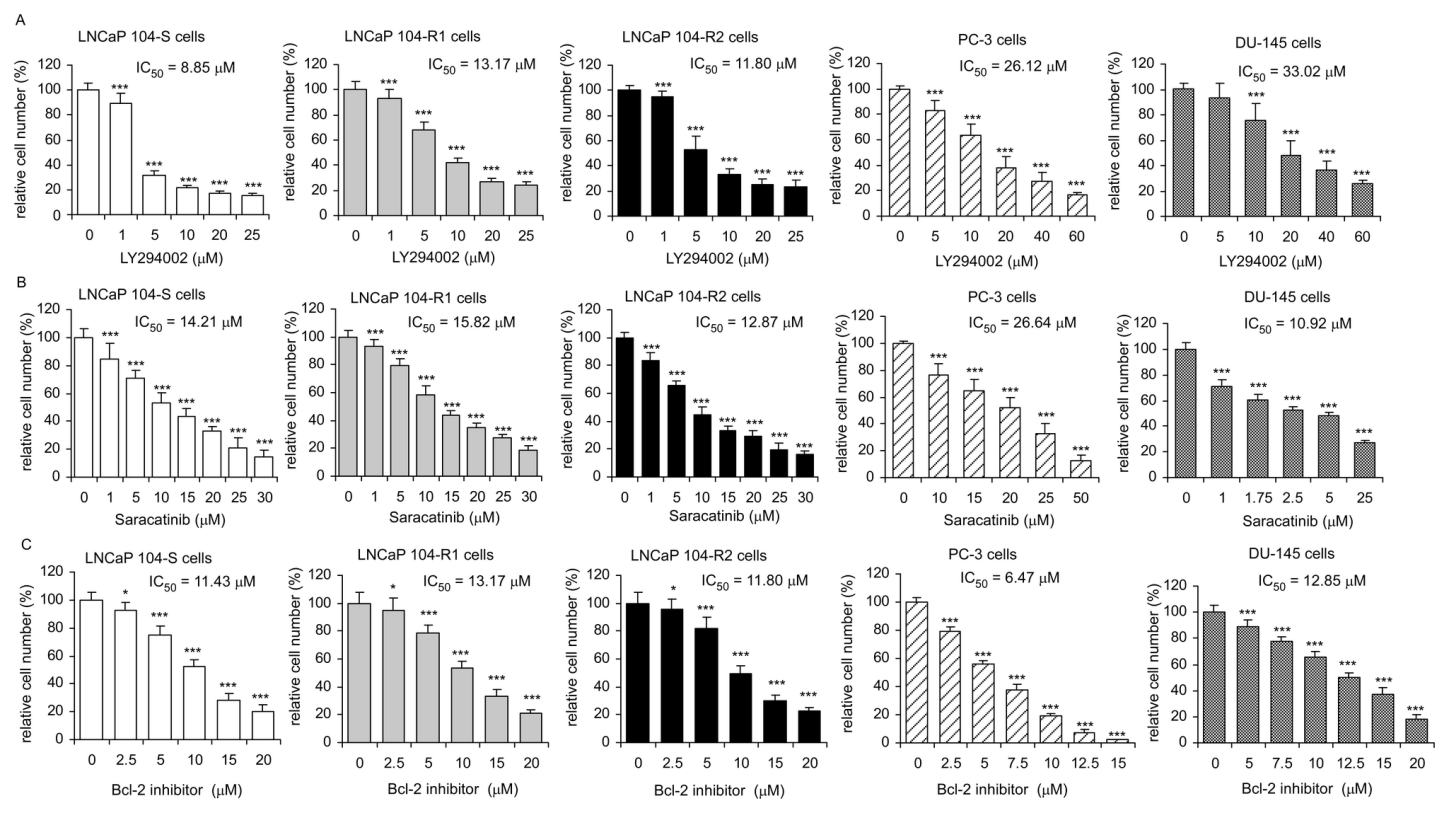
Effect of LY294002, Saracatinib, or ABT 737 on proliferation of LNCaP, PC-3, and DU-145 cell lines. LNCaP 104-S, 104-R1, 104-R2, PC-3, and DU-145 cells were treated with increasing concentrations of LY294002 (A), Saracatinib (B), or ABT 737 (C) for 72 hrs. Relative cell number of LNCaP cells was determined by Hoechst dye 33258-based 96-well proliferation assay as described in Materials and Methods. Cell numbers were normalized to control (dimethylsulfoxide) of each cell line. Triple asterisks (***) represent statistically significant difference *p* <0.001 between the treated group and the control group.

IC_50_ from Figures 7-10 was used for calculation of ratio. IC_50_ of 104-S cells for all different inhibitors was set to 1 for comparison.

### Sensitivity of LNCaP sublines, DU-145, and PC-3 cells to kinase inhibitors and Bcl-2 inhibitor

Compared to LNCaP 104-S cells, 104-R1 and 104-R2 cells were more resistant to the treatment of PI3K inhibitor LY294002 and EGFR inhibitor AG1478 (Figure 9, Figure 10, Table 2). Sensitivity of 104-R1 and 104-R2 cells to EGFR inhibitor Gefitinib and Bcl-2 inhibitor ABT 737 was similar to that of 104-S cells. 104-R2 cells were slightly more sensitive to treatment of Src inhibitor Saracatinib. Compared to 104-S cells, PC-3 and Du-145 cells were more resistant to most drugs being tested. However, PC-3 cells were more sensitive to treatment of AG1478 or ABT 737 while DU-145 cells were more sensitive to Saracatinib.

### Overexpression of Skp2 elevated resistance of 104-S and 104-R1 cells to chemotherapy drugs treatment

**Figure 11:**
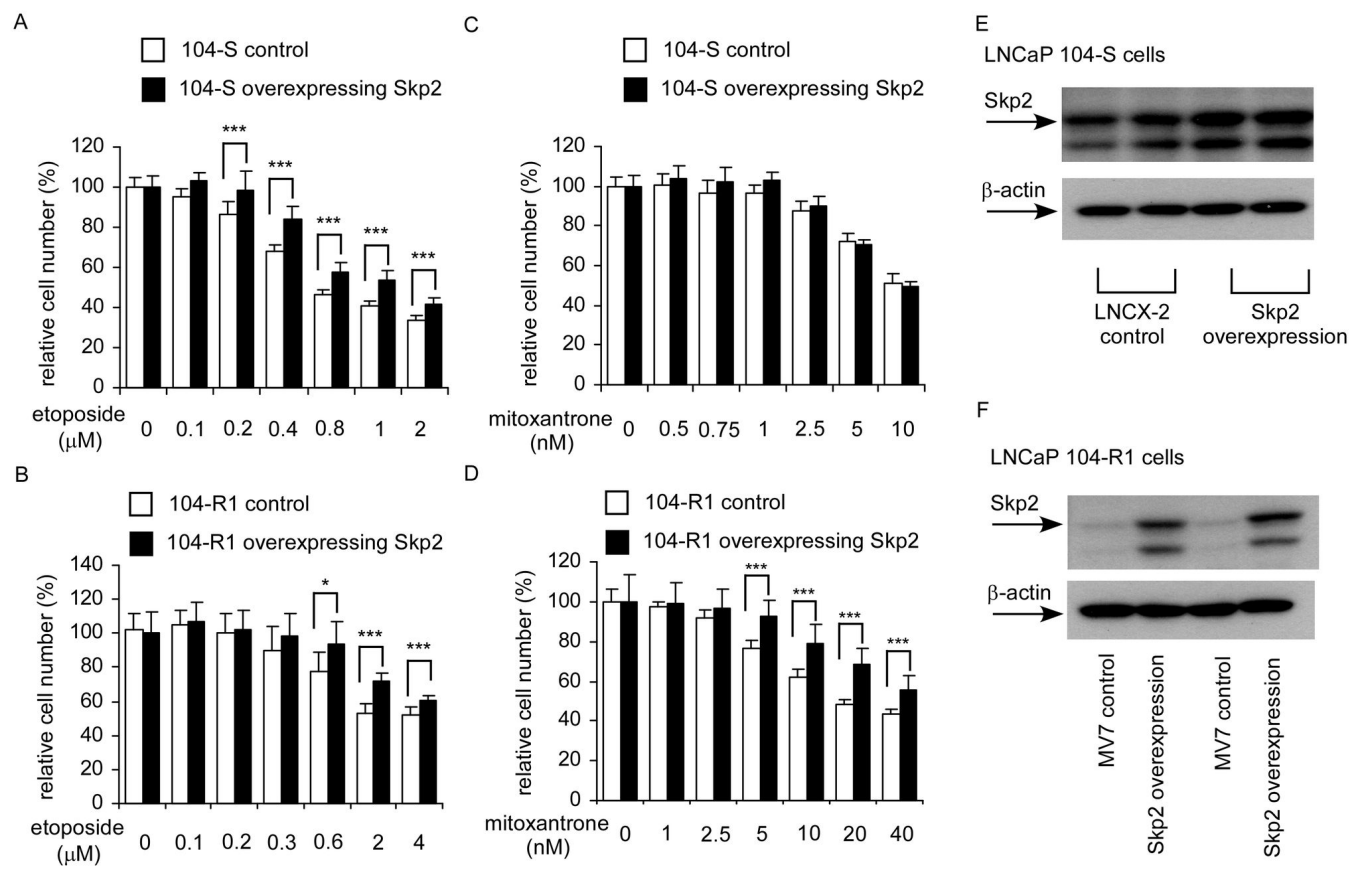
Effect of Skp2 overexpression on sensitivity of LNCaP cells to chemotherapy drug treatment. LNCaP 104-S, 104-S overexpressing Skp2, 104-R1, and 104-R1 overexpressing Skp2 cells were treated with increasing concentrations of etoposide (A, B) or mitoxantrone (C, D) for 72 hrs. Relative cell number of LNCaP cells was determined by Hoechst dye 33258-based 96-well proliferation assay as described in Materials and Methods. Cell numbers were normalized to control (dimethylsulfoxide) of each cell line. Triple asterisks *** represent statistically significant difference *p* <0.05 and *p* <0.001, respectively, between the treated group and the control group. Overexpression of Skp2 protein in 104-S (E) and 104-R1 (F) cells was confirmed by Western blotting.

As 104-R1 and 104-R2 cells expressed higher c-Myc and Skp2 proteins as compared to 104-S cells (Figure 4), we determined if overexpression of c-Myc or Skp2 may increase resistant of LNCaP cells to chemotherapy drugs. Overexpression of Skp2 increased resistance of 104-S cells to etoposide treatment and resistance of 104-R1 cells to both etoposide and mitoxantrone treatments (Figure 11). However, overexpression of c-Myc did not increase drug resistance of either 104-S or 104-R1 cells (data not shown). 

## Discussion

Phosphatase and tensin homolog (PTEN) protein is a phosphatase dephosphorylating phosphatidylinositol (3–5)-trisphosphate. PTEN is a negative regulator for phosphoinositide 3-kinase/Akt signaling pathway [31]. PTEN is frequently deleted or mutated in prostatic intraepithelial neoplasia (PIN) and prostate cancer, resulting in up-regulation of PI3K/Akt signaling[32,33]. PI3K/Akt signaling plays an important role in survival of prostate cancer cells [32]. Up-regulation of PI3K/Akt activity is associated with poor clinical outcome of prostate cancer [33-39]. Akt is a serine/threonine protein kinase regulating a variety of cellular responses, including inhibition of apoptosis and stimulation of cellular proliferation [40,41]. There are three mammalian isoforms, Akt1, Akt2, and Akt3. Akt1 is involved in cellular survival pathways via inhibiting apoptotic processes [40,41]. Two phosphorylation sites on Akt, threonine 308 and serine 473, regulate activity of Akt. Phosphorylation of Thr308 on Akt is activated by PDK1 [42]. Phosphorylation of serine 473 on Akt is activated by mTOR kinase, its associated protein rector, and SIN1/MIP1 [43,44]. Level of phospho-Akt correlates with higher Gleason score, immunoreactivity for Ki67, and level of phospho-EGFR [45,46]. LNCaP cells were previously being reported to constitutively express Akt and phospho-Akt [47]. Researchers found that the expression level of Akt and phospho-Akt was not different between androgen-dependent parental cell line and the long-term androgen-ablated LNCaP sublines [47]. However, we observed a lower protein abundance of Akt1 and phospho-Akt T308 in 104-R1 and 104-R2 cells than those in 104-S cells. The medium for proliferation assay is similar to their culture medium, which equals DMEM plus 10% FBS and 0.1 nM R1881 for 104-S cells and DMEM plus 10% CS-FBS and no androgen for 104-R1 and 104-R2 cells. Under this condition, 104-R1 and 104-R2 cells express a similar ratio of phospho-Akt Thr308/Akt and a higher ratio of phospho-Akt Ser473/Akt as compared to 104-S cells (Table 1). This suggested the possibility that Akt signaling plays an essential role for proliferation or survival in 104-R1 and 104-R2 cells. It has been reported that long-term androgen-ablated LNCaP sublines are relatively resistant to induction of cell death by LY294002 or the etoposide [47]. We observed that 104-R1 and 104-R2 cells are slightly more resistant to treatment of LY294002 (Figure 10). Surprisingly, PC-3 and DU-145 expressed very low level of Akt phosphorylation, suggesting that Akt probably does not play major role in these two cells (Figure 5). In consistent with this hypothesis, PC-3 and DU-145 cells were very resistant to treatment with LY294002. 

The 104-R1 and 104-R2 cells express much less EGFR protein than 104-S cells, suggesting that EGFR signaling overall is significantly decreased during progression from androgen-dependent status to androgen-independent status of LNCaP cells under androgen ablation therapy (Figure 5). Interesting, the 104-R1 and 104-R2 cells express phospho-EGFR protein at comparable or higher levels as those in 104-S cells (Figure 5, Table 1). The ratio of phospho-EGFR/EGFR of different tyrosine sites in 104-R1 and 104-R2 cells was approximately 3-10 fold and 15-30 folds higher than that of 104-S cells, respectively (Table 1). This finding suggested the possibility that phosphorylation of tyrosines on EGFR play important roles in regulating proliferation or survival of 104-R1 and 104-R2 cells. EGFR is the receptor for the epidermal growth factor (EGF) and a member of the ErbB family of receptor tyrosine kinase (RTK) which plays essential role in regulating cell proliferation and signaling transduction [48,49]. Activating EGFR with EGF induces autophosphorylation, which results in a downstream cascade that leads to increased cellular proliferation [50]. Autocrine activation of EGFR is a common feature of prostate cancer cells in contrast to normal prostate epithelial cells [51]. EGFR signaling can activate AR through phosphorylation in the absence of the androgen and PI3K/Akt signaling [50]. The cross-talk between EGFR and AR is important in regulating proliferation of androgen-independent prostate cancer cells [50,52-54]. LNCaP 104-R1 and 104-R2 are AR-rich androgen-independent cells growing in medium without androgens. Up-regulation of EGFR activity in 104-R1 and 104-R2 cells may activate downstream AR signaling and promote cell survival and proliferation in the absence of androgen. Inhibition of EGFR signaling using Gefitinib (Iressa), a tyrosine kinase inhibitor, causes G1 cell cycle arrest and growth inhibition in prostate cancer cells [55,56]. Our observation indicated that 104-R1 and 104-R2 cells were as sensitive as 104-S cells to treatment with Gefitinib. PC-3 and DU-145 expressed higher phosphorylation level of Tyr1045 and Tyr845 on EGFR as compared to 104-S cells (Figure 5). PC-3 cells showed less phosphorylation on other tyrosine sites on EGFR. However, PC-3 cells was more sensitive to treatment with EGFR inhibitor AG1478 (Figure 9), suggesting a possibility of using AG1478 as treatment for prostate cancer bone metastasis. AR and EGFR are negative correlated to each other in LNCaP [57,58]. In progression models developed by other group or our group [7-9,11,12,14,59], AR elevation is essential and necessary. It is possible that down-regulation of EGFR in 104-R1 and 104-R2 cells (Figure 5) is a necessary step for elevation of AR. 

Androgen receptor (AR) is an androgen-activated transcription factor and belongs to the nuclear receptor super family. Binding of DHT to the androgen receptor (AR) induces dissociation of AR from heat-shock proteins (HSPs) and stimulates AR phosphorylation [60]. AR dimerizes, translocates into the nucleus, and binds to androgen-response elements (ARE) in the promoter regions of target genes [60]. Based on gene microarray studies of seven different human prostate cancer xenograft models, the increase of AR mRNA is the only change consistently associated with the development of the castration-resistant prostate cancers [59]. Elevated AR expression in hormone-refractory prostate cancer cells or recurrent hormone-refractory tumors is observed in our progression model [7-9,11,12,61] and by several other groups [59,62-74]. We observed that AR mRNA increases 2 fold while AR protein increases 1.6-1.8 fold during the progression from LNCaP 104-S to 104-R1 and 104-R2 status (Figure 2A, Figure 4). The higher AR expression levels in 104-R1 and 104-R2 cells may be responsible for the higher induction of PSA mRNA and protein following androgen treatment in these cells (Figure 2B, Figure 4). The observations that castration-resistant 104-R1 and 104-R2 cells express PSA mRNA and protein in the absence of androgen 2-fold higher than that in 104-S cells may be similar to the clinical situation where hormone refractory prostate cancer develops in patients after androgen deprivation therapy. The serum levels of PSA in these patients increase several fold while the serum testosterone level is very low due to the androgen ablation therapy [75,76]. Activation of protein kinase A (PKA) in the absence of androgen has also been reported to activate AR and to stimulate production of PSA [77], which may explain our observation. Additionally, it has been reported that IL-8 signaling increases AR expression and promotes ligand-independent activation of AR in LNCaP cells [78]. IL-8 signaling may also contribute to the activation of AR in LNCaP 104-R1 and 104-R2 cells. LNCaP cells express a mutant AR (T877A) that displays relaxed ligand binding specificity [29,30]. We did not find any additional mutation in AR ligand binding domain in 104-R1 and 104-R2 cells (Figure 3).

Androgen treatment at physiological concentration significantly suppressed the proliferation of 104-R1 and 104-R2 cells via induction of G1 cell cycle arrest (Figure 1). The 104-R2 cells were slightly more sensitive to androgen treatment as compared to 104-R1 cells. S-phase kinase-associated protein 2 (Skp2) is a member of the F-box protein family which is responsible for ubiquitination and down-regulation of p27^Kip^ [79,80]. The p27^Kip^ is a cell cycle inhibitory proteins and elevation of p27^Kip^ can result in cell cycle arrest and reduction of cell proliferation in prostate cancer cells [40]. The c-Myc protein, a well-known proto-oncoprotein, is an important transcriptional regulator of the androgenic response and cell proliferation in prostate cancer [7,81]. Expression of c-Myc mRNA and protein both increase during progression of prostate cancer [40]. Decrease in c-Myc abundance suppresses cell proliferation and tumor growth of prostate cancer cells [40]. Androgen treatment reduces c-Myc and Skp2 expression as well as increases p27^Kip^ abundance, which results in G1 cell cycle arrest in 104-R1 and 104-R2 cells [14,22]. The androgen-suppressive phenotype of AR-positive hormone-refractory LNCaP cells has been observed by other groups [8,82-85]. PC-3 is a commonly used AR-negative human prostate cancer cell line established from a bone-derived metastasis [86]. Physiological concentrations of DHT cause growth inhibition, G1 cell cycle arrest, and apoptosis in PC-3 cells overexpressing full length wild-type AR [87-89]. Androgen treatment has also been reported to suppress growth of other prostate cancer cell lines, including ARCaP and MDA PCa 2b-hr [70,90]. These observations suggested that AR functions as a tumor suppressor in advanced prostate cancer cells when it is expressed at high levels and is being activated by androgen[13]. Manipulating androgen/AR signaling can be a potential therapy for AR-positive advanced prostate cancers [13].

Mitogen-activated protein kinases (MAPK) are serine/threonine-specific protein kinases. MAPKs are involved in directing cellular responses to mitogens, osmotic stress, heat shock, and proinflammatory cytokines [91]. Constitutive activation of MAPK/ERK signaling inhibits proliferation of prostate cancer cells via upregulation of BRCA2 [92]. We observed a reduction of phosphorylation of ERK1/2 in 104-R1 and 104-R2 cells as compared to 104-S cells (Figure 5, Table 1), which may provide growth advantage for 104-R1 and 104-R2 cells. Forced expression of fatty acid synthase (FAS) promoted proliferation while siRNA knockdown of FASN induced apoptosis in prostate cancer cells [40]. However, protein expression of FAS is not significantly different in 104-S, 104-R2, and 104-R2 cells (Figure 5).

Interleukin-8 (IL-8) has been reported to activate the AR and to promote androgen-independent growth via Src and FAK (focal adhesion kinase) signaling [93]. Treatment with Src inhibitors suppresses IL-8-induced cell migration and androgen-independent growth [93]. This result suggests that both growth and migration of prostate cancer cells depend on the activity of Src, whereas cell migration also requires the activation of FAK [93]. Neuropeptide bombesin can activate AR and confer androgen-independent growth of prostate cancer cells [94]. Src kinase and its target gene c-Myc are critical for bombesin-induced AR-mediated activity and are required for translocation and transactivation of AR [40]. Src inhibitor treatment inhibits proliferation of prostate cancer cells through β-catenin, ERK1/2, GSK3β-mediated cyclin D1, and c-Myc regulation [95]. Src inhibitor also inhibits cell motility via suppression of FAK, p130CAS, and paxillin activation [95]. Administration of Src inhibitor AZD0530 in mice reduced orthotopic DU-145 xenograft growth by 45% [95]. Our result suggested that Src inhibitor can effectively suppress the proliferation of androgen-independent 104-R1 and 104-R2 cells as well as DU-145 cells (Figure 10). The higher resistance of PC-3 cells to treatment with Saracatinib was probably due to the higher level of Src phosphorylation in PC-3 cells (Figure 5).

Bcl-2 is an anti-apoptotic oncoprotein. Normal human prostate epithelial cells do not express the bcl-2 protein [96]. Overexpression of Bcl-2 protects LNCaP prostate cancer cells from apoptosis and confers resistance to androgen ablation treatment [96]. Ectopic expression of the Bcl-2 antagonist Bax or Bcl-2 shRNA the retards the progression from androgen-dependent to androgen-independent status of LNCaP cells growing in androgen depleted medium [97]. Up-regulation of Bcl-2 is necessary for the progression of LNCaP prostate cancer cells from an androgen-dependent to an androgen-independent growth stage [97]. In our observation, Bcl-2 protein was barely detectable in 104-S cells, while androgen treatment suppressed protein expression of Bcl-2 in 104-R1 and 104-R2 cells. Since silencing of Bcl-2 suppresses the growth of LNCaP 104-R1 xenograft in nude mice [97], reduction of Bcl-2 by R1881 in 104-R1 and 104-R2 cells may partially contribute to the growth inhibition caused by androgen treatment. Our observation indicated that Bcl-2 inhibitor can effectively suppressed 104-R1 and 104-R2 cells (Figure 10). Surprisingly, 104-S cells also respond to treatment of Bcl-2 inhibitor (Figure 10). Prostate tumors of advancing stage are associated with a significant increase in P53 expression [98]. Elevation of protein expression of Bcl-2 and P53 appear to be important biomarkers to predict recurrence and poor survival in patients with clinically localized prostate cancer after radical prostatectomy [99-103]. Protein level of Bcl-2 and P53 proteins increases after androgen ablation therapy in prostate cancer patients [104,105]. We observed higher protein expression levels of Bcl-2 and P53 in 104-R1 and 104-R2 cells as compared to 104-S cells (Figure 4). The fact that we observed that androgen treatment lowered the protein expression of BCl-2 and P53 in 104-R1 and 104-R2 cells (Figure 4) may strengthen the rationale of using androgen as a treatment for advanced prostate cancers. Androgens have been reported to repress Bcl-2 expression via activation of the retinoblastoma (RB) protein in prostate cancer cells [40]. The retinoblastoma (RB) protein plays a critical role in androgen regulation of Bcl-2 [106]. The phosphorylation levels of RB at serine residues 780 and 795 were decreased in LNCaP cells treated with androgens [40]. Androgens treatment decreases phosphorylation of Rb and increases cyclin-dependent kinase inhibitors (CDKI) P15^INK4B^ and P27^Kip1^[40]. Androgens activate the CDKI-Rb axis, which negatively modulates activities of the E2F site in the Bcl-2 promoter, and thus suppresses Bcl-2 expression [106]. Our observation is consistent to this previous finding. We observed that androgen reduces phosphorylation and abundance of Rb, as well as reduces expression of Bcl-2 (Figure 4). LNCaP 104-S, 104-R1, 104-R2, and DU-145 cells showed similar sensitivity to BCl-2 inhibitor ABT 737 while PC-3 cells exhibit greater sensitivity to this inhibitor (Table 2). Bcl-2 inhibitor is thus a potential treatment for prostate cancer progression or metastasis. 

We observed that androgen treatment suppressed protein expression of P53 in LNCaP 104-R1 and 104-R2 cells (Figure 4). Previously, Ma et al., reported that androgen treatment stabilizes AR, and AR directly suppresses miR-29a expression [107]. The miR-29 miRNAs directly suppress Cdc42 and p85α, resulting in the stabilization of P53. P53 then inhibits the transcription of AR [107,108]. Therefore, the reduction of P53 by androgen treatment may be correlated to the induction of AR in LNCaP 104-R1 and 104-R2 cells.

Etoposide, vinblastine, paclitaxel, and motoxantrone have previously been shown to suppress the proliferation of LNCaP, 22Rv1, and PC-3 prostate cancer cells [40]. In our study, the androgen-independent 104-R1 and 104-R2 cells are more resistant to the treatment of all chemotherapy drugs and inhibitors tested in this study, revealing the difficulty of treatment of castration-resistant prostate cancers. The 104-R1 and 104-R2 cells were especially more resistant to etoposide and mitoxantrone, both with IC_50_ 2-3.5 fold higher than that of 104-S cells (Table 2). Etoposide treatment dose-dependently inhibited AR-mediated cell growth, AR transcription, AR nuclei translocation, binding of androgen to AR, AR mRNA and protein expression level, and production of PSA in LNCaP prostate cancer cells [109]. The fact that the proliferation of 104-S cells is androgen-dependent while proliferation of 104-R1 and 104-R2 cells is androgen-independent might explain why 104-S cells are more sensitive to etoposide treatment. We therefore believe that paclitaxel and vinblastine might be more effective for treating androgen-independent relapsed prostate tumors. Compared to 104-R1 cells, 104-R2 cells are more sensitive to the treatment of chemotherapy drugs and inhibitors except vinblastine (Table 2). LNCaP 104-R2 cells evolve from androgen-dependent 104-S cells after 18-24 months of androgen depletion culture [8,9,14]. Our observation suggested that chemotherapy drugs or inhibitor targeting either Akt or EGFR can still be effective to treat advanced prostate cancer even the patient has received androgen ablation therapy for more than 1-2 years. 

In conclusion, our observations indicated targeting AR, PI3K/Akt, Bcl-2, Src, and EGFR signaling pathway may be a choice for treatment of castration-resistant AR-positive prostate cancers.
